# Screening for *EGFR* Amplifications with a Novel Method and Their Significance for the Outcome of Glioblastoma Patients

**DOI:** 10.1371/journal.pone.0065444

**Published:** 2013-06-06

**Authors:** Michał Bieńkowski, Sylwester Piaskowski, Ewelina Stoczyńska-Fidelus, Małgorzata Szybka, Mateusz Banaszczyk, Monika Witusik-Perkowska, Emilia Jesień-Lewandowicz, Dariusz J. Jaskólski, Anna Radomiak-Załuska, Dorota Jesionek-Kupnicka, Beata Sikorska, Wielisław Papierz, Piotr Rieske, Paweł P. Liberski

**Affiliations:** 1 Department of Molecular Pathology and Neuropathology, Chair of Oncology, Medical University of Lodz, Lodz, Poland; 2 Department of Radiotherapy, Chair of Oncology, Medical University of Lodz, Lodz, Poland; 3 Department of Neurosurgery, Medical University of Lodz, Lodz, Poland; 4 Ward of Neurosurgery, Maria Sklodowska-Curie Hospital in Zgierz, Zgierz, Poland; 5 Department of Pathology, Chair of Oncology, Medical University of Lodz, Lodz, Poland; 6 Department of Pathomorphology, Medical University of Lodz, Lodz, Poland; The University of Hong Kong, Hong Kong

## Abstract

Glioblastoma is a highly aggressive tumour of the central nervous system, characterised by poor prognosis irrespective of the applied treatment. The aim of our study was to analyse whether the molecular markers of glioblastoma (*i.e. TP53* and *IDH1* mutations, *CDKN2A* deletion, *EGFR* amplification, chromosome 7 polysomy and *EGFRvIII* expression) could be associated with distinct prognosis and/or response to the therapy. Moreover, we describe a method which allows for a reliable, as well as time- and cost-effective, screening for *EGFR* amplification and chromosome 7 polysomy with quantitative Real-Time PCR at DNA level. In the clinical data, only the patient’s age had prognostic significance (continuous: HR = 1.04; p<0.01). At the molecular level, *EGFRvIII* expression was associated with a better prognosis (HR = 0.37; p = 0.04). Intriguingly, *EGFR* amplification was associated with a worse outcome in younger patients (HR = 3.75; p<0.01) and in patients treated with radiotherapy (HR = 2.71; p = 0.03). We did not observe any difference between the patients with the amplification treated with radiotherapy and the patients without such a treatment. Next, *EGFR* amplification was related to a better prognosis in combination with the homozygous *CDKN2A* deletion (HR = 0.12; p = 0.01), but to a poorer prognosis in combination with chromosome 7 polysomy (HR = 14.88; p = 0.01). Importantly, the results emphasise the necessity to distinguish both mechanisms of the increased *EGFR* gene copy number (amplification and polysomy). To conclude, although the data presented here require validation in different groups of patients, they strongly advocate the consideration of the patient’s tumour molecular characteristics in the selection of the therapy.

## Introduction

Glioblastoma is the most common tumour of the central nervous system in adults with annual occurrence of about 3 per 100,000 population [1]. As a highly aggressive neoplasm, it is characterised by the median survival of untreated patients of about 3 months. Neurosurgery prolongs the survival to about 10 months and the following temozolomide-based radio-chemotherapy (currently the standard treatment) to about 15 months [2], [3]. Although numerous novel therapeutic methods are being introduced, only targeting integrins was reported to extend the survival of glioblastoma patients to more than 20 months [4]. Taking into account the high heterogeneity of glioblastoma, it is reasonable to assume that certain molecular subtypes may be characterised by a different response to distinct therapies. To date, this has been shown only for the classical subtype (according to Verhaak’s classification) as well as for the *MGMT* promoter methylation in the temozolomide-based radio-chemotherapy [5], [6]. Additionally, the molecular characteristics may be potentially informative with regards to the prognosis; however, no molecular marker has been unanimously validated in glioblastoma for that purpose. The clinical factors of the recognised prognostic value are the age and the general condition of the patient (both included in the Radiation Therapy Oncology Group, RTOG, classification) as well as the extent of neurosurgical resection [5], [7]. The aim of this article is to analyse the impact of several molecular alterations characteristic for glioblastoma (*i.e. TP53* and *IDH1* mutations, *CDKN2A* deletion, *EGFR* amplification, chromosome 7 polysomy and *EGFRvIII* expression) in patients treated with a neurosurgical operation and with or without the following therapy (radiotherapy or radio-chemotherapy).

## Materials and Methods

### 1. Analysed Group/Clinical Data

The analysed group consists of 83 glioblastoma patients who underwent a neurosurgical resection of the tumour at Norbert Barlicki University Clinical Hospital No. 1 in Łódź and at Maria Skłodowska–Curie Provincial Specialist Hospital in Zgierz. Tumour and blood samples for molecular analyses as well as clinical data were obtained according to protocols approved by the ethical committee of Medical University of Łódź. Written informed consent was obtained from all patients and their data were processed and stored according to the principles expressed in the Declaration of Helsinki. All patients were diagnosed with glioblastoma WHO grade IV by a neuropathologist. None of the patients had an earlier diagnosis of astrocytoma, thus, all cases were regarded as primary glioblastoma. Karnofsky Performance Status (KPS and, hence, RTOG classification) data were not available for the most of the patients and were not included in our analysis. The clinical data gathered for this project included: the age of the patient at the time of the diagnosis, sex, location of the tumour, the extent of resection, the following therapy (radiotherapy or radio-chemotherapy) and the overall survival time. The patients were aged from 23 to 84 years (the median age was 60 years), the M:F ratio was 1.18. The survival times were available for 60 patients (51 complete and 9 censored responses) and varied between 1 and 48 months (the median survival was 10 months).

### 2. DNA/RNA Isolation and Reverse Transcription

Total cellular DNA and RNA were isolated from non-marginal fragments of frozen tumour samples (stored at -80°C) and frozen peripheral blood leukocytes obtained from the patients using AllPrep DNA/RNA Mini Kit (Qiagen, Germany) according to the manufacturer’s protocol. RNA samples were treated with DNase following the isolation. RNA and DNA concentrations were measured spectrophotometrically. The content of tumour cells in each sample was estimated as >70%, according to the STR analysis performed as described earlier [8]. 100 ng of total RNA was reverse transcribed into a single-stranded cDNA in a 20 µl reaction volume using QuantiTect Rev. Transcription Kit (Qiagen, Germany) according to the manufacturer’s protocol.

### 3. Novel Method of the *EGFR* Gene Analysis by Quantitative Real-Time PCR at the DNA Level

**Figure 1:**
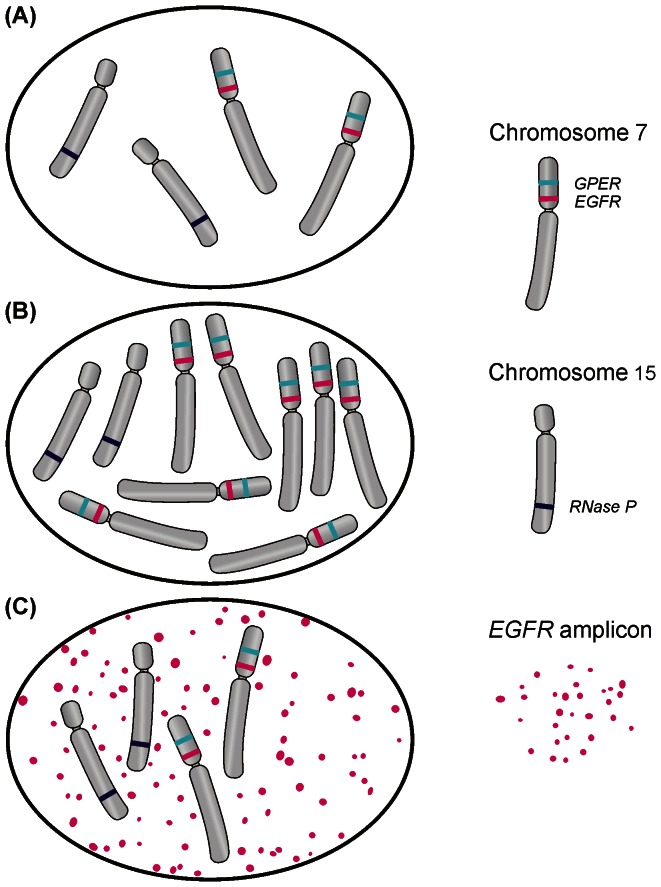
A diagram depicting the premises upon which the *EGFR* gene copy number analysis is based. In normal cells both the ratio of *EGFR* to *GPER* and the ratio of *GPER* to *RNase* is equal to 1. In cells with chromosome 7 polysomy the ratio of *GPER* to *RNase* increases, while in cells with *EGFR* amplification the ratio of *EGFR* to *GPER* increases. In cells with both the polysomy and the amplification both ratios are increased and the ratio of *EGFR* to *RNase* is equal to their product. A. normal cell; B. cell with chromosome 7 polysomy; C. cell with extrachromosomal *EGFR* amplification.

To determine the *EGFR* gene dosage level in each sample quantitative Real-Time PCR was performed using Rotor-Gene 6000 system (Qiagen, Germany). Each sample was amplified in triplicate in a 10 µl reaction volume containing 10 ng of DNA, a 1x reaction mixture containing Syto9 (Life Technologies, US) and 35 ng each of the forward and reverse primers. The cycling conditions for the Real-Time PCR reactions were as follows: 3 min at 95°C (polymerase activation) followed by 40 cycles of 20 s at 95°C (denaturation), 30 s at 60°C (annealing) and 20 s at 72°C (extension). All primer sequences are listed in Table S1. The gene dissociation curve was analysed for each sample to confirm the specificity of the amplification signal. The normalised relative gene dosage level of the tested samples compared to the control sample was calculated using the method previously described by Pfaffl *et al.* based on each sample’s average CT value and each gene’s average PCR efficiency [9]. DNA derived from non-tumorous tissue (peripheral blood leukocytes) was used as a control and the gene dosage in normal tissue was assumed to be 1. The technique presented is based on the assumption that the ratio of *EGFR* to the other marker, located within chromosome 7, would be equal to 1 if there are no amplicons (assuming no LOH within either of the analysed loci). The selection of the chromosome 7 marker was based on two criteria. Firstly, the locus of the marker had to be located within the same chromosomal arm at an appropriate distance from the *EGFR* gene locus (7p12) in order to minimise the probability of its inclusion into the amplicons [10], [11]. Secondly, the locus of the marker had to be located within a region which is retained in gliomas. These criteria were fulfilled by the *GPER* gene (7p22) [12], [13]. The following method of result interpretation was applied: the cumulative *EGFR* gene dosage was assessed by the ratio of *EGFR* to *RNaseP* (the *RPP25* gene located within chromosomal region 15q24.2) [14], [15]; chromosome 7 polysomy was identified when the ratio of *GPER* to *RNaseP* was higher than 1.5; while *EGFR* amplification was identified when the ratio of *EGFR* to *GPER* was higher than 1.5 (Fig. 1).

### 4. Standard Analysis of the *EGFR* Gene Dosage by Quantitative Real-Time PCR at the DNA Level

For the comparative purposes, the standard method of the *EGFR* gene copy number assessment with quantitative Real-Time PCR has been applied. The reactions for *EGFR* and *RNaseP* were performed as explained in point 2.3. and the ratio of *EGFR* to *RNaseP* was calculated analogously. The results were interpreted in the following manner: the ratio of *EGFR* to *RNaseP* between 1.5 and 5 was considered as resulting from the polysomy, while the ratio of *EGFR* to *RNaseP* higher than 5 was considered as resulting from the amplification.

### 5. Fluorescence *in situ* Hybridization (FISH)

FISH was performed with FISH Pretreatment Reagent Kit (Abbott Molecular, US) according to the manufacturer’s protocol. In brief, a commercial probe set (Vysis LSI EGFR SpectrumOrange/CEP 7 SpectrumGreen; Abbott Molecular, US) was used to simultaneously detect the copy numbers of the *EGFR* gene and of chromosome 7. FISH was performed using the following procedure: the fixed slides were incubated in 2x standard saline citrate (SSC) at 72°C for 5 min, immersed in protease solution for 10 min at 37°C, washed with PBS for 5 min at room temperature, fixed with 1% formaldehyde for 5 min at room temperature, washed in PBS for 5 min at room temperature, dehydrated in 70%, 85% and 100% ethanol for 1 min each, then air dried and placed on a 50°C slide warmer for 2 min. The FISH probe mix was centrifuged and denatured at 73°C for 5 min. The denatured probe was added to each specimen. The slides were then coverslipped and incubated at 37°C overnight in a humidified chamber. Next, the slides were washed with 0.4×SSC/0.3% NP-40 at 73°C for 2 min, rinsed in 2×SSC/0.1% NP-40 for 1 min at room temperature and air dried in darkness. Before coverslipping, 10 µl of DAPI II counterstain was added to the slides. To score the samples, an Olympus BX-41 fluorescence microscope equipped with a specially designed filter combination for green and orange spectra was used. The number of red signals, caused by the binding of the *EGFR*-specific probe, directly reflects the number of copies of *EGFR*. The number of green signals, caused by the binding of the CEP 7 probe, directly reflects the number of copies of chromosome 7. FISH evaluation was performed using previously published criteria [16]. For each sample at least 100 nuclei were analysed. The EGFR/CEP 7 ratio was calculated and samples containing three or more signals specific for CEP 7 per nucleus were defined as having chromosome 7 polysomy. Samples with intrachromosomal amplification ratios of 2 or greater were considered to be amplified for *EGFR*. Extrachromosomal amplification of *EGFR* was defined as the presence of at least three times as many *EGFR* signals as centromere 7 signals per cell [17]. An exemplary FISH result is presented in Fig. S1A.

### 6. FISH in Formalin-Fixed, Paraffin-Embedded (FFPE) Samples

FISH in FFPE samples processed for routine histopathology was performed with Paraffin Pretreatment Reagent Kit (Abbott Molecular, US) according to the manufacturer’s protocol. In brief, a commercial probe set (Vysis LSI EGFR SpectrumOrange/CEP 7 SpectrumGreen; Abbott Molecular, US) was used to simultaneously detect the copy numbers of the *EGFR* gene and of chromosome 7. FISH was performed using the following procedure: the slides were deparaffinised in xylene two times for 20 min at room temperature, rehydrated in a 100%, 80% and 70% ethanol for 1 min each at room temperature and air dried on 50°C slide warmer. Then, the slides were immersed in 0,2N HCl for 20 min at room temperature, washed with purified water and wash buffer for 3 min each at room temperature, incubated in pretreatment solution for 30 min at 80°C and washed in purified water for 1 min at room temperature and in wash buffers two times for 5 min at room temperature. Next, the slides were incubated in protease solution for 30 min at 37°C, washed in wash buffers two times for 5 min at room temperature and air dried on 50°C slide warmer. Then, the slides were fixed in 10% buffered formalin for 10 min at room temperature, washed in wash buffers two times for 5 min at room temperature and air dried on 50°C slide warmer. The FISH probe mix was centrifuged and denatured at 73°C for 5 min. The denatured probe was added to each specimen. The slides were then coverslipped and incubated at 37°C overnight in a humidified chamber. Next, the slides were washed with 0.4×SSC/0.3% NP-40 at 73°C for 2 min, rinsed in 2×SSC/0.1% NP-40 for 1 min at room temperature and air dried in darkness. Before coverslipping, 10 µl of DAPI II counterstain was added to the slides. The evaluation was performed with confocal laser scanning microscope Olympus FluoView1000 according to the criteria mentioned in point 2.5. Exemplary FISH in FFPE sample results are presented in Fig. S1BC.

### 7. *TP53* and *IDH1* Sequencing Analysis

Exons 5–8 of the *TP53* gene and exon 4, including codon 132, of the *IDH1* gene were amplified by PCR on cDNA template and sequenced using the dideoxy termination method and SequiTherm Excel DNA Sequencing Kit (Epicentre Technologies) following the manufacturer’s protocol. The primer sequences are listed in Table S1. LiCor automatic sequencer system was applied to the separation and analysis of PCR-sequencing products. To verify the results of sequencing the semiquantitative densitometric analysis was performed. The intensity of wild-type and mutated bands was estimated by comparison to the neighbouring bands in the same sequencing lane used as a reference. The results of the *TP53* sequencing have been deposited in GenBank (Accession Numbers: KC820708-KC820786). Exemplary sequencing results are presented in Fig. S1D.

### 8. Detection of the *CDKN2A* Deletions at the DNA Level by Quantitative Real-Time PCR

To determine the *CDKN2A* exon 1 and/or exon 2 deletions quantitative Real-Time PCR reactions were performed as described above. The primer sequences are listed in Table S1. The reference gene was *RNaseP*. DNA derived from non-neoplastic tissue (leukocytes) was used as a control and the gene dosage in normal tissue was assumed to be 1. Each sample was analysed three times. An average value lower than 0.5 was considered to represent the deletion of the tested gene in the general population of cells. *CDKN2A* exon 1 and/or exon 2 deletion was confirmed by agarose gel electrophoresis using BioRad Quantity One 1-D Analysis Software.

### 9. Detection of the *EGFRvIII* Expression at the cDNA Level by Quantitative Real-Time PCR

To determine the *EGFRvIII* expression quantitative Real-Time PCR reactions were performed as described above. The *EGFRvIII*-specific primers were based on a previous report [18], the primer sequences are listed in Table S1. *GUSB* was used as a reference gene for the normalization of the target gene expression level. To evaluate the *EGFRvIII* expression, cDNA derived from tumour tissue positive for *EGFRvIII* was used as a control. The Real-Time PCR was preceded by a conventional RT-PCR applied to examine the tested tumour samples in terms of *EGFRvIII* expression.

### 10. Statistical Analysis

Statistical analyses were performed using STATISTICA 10 software (StatSoft, US). In order to assess the significance of given feature a series of analyses was performed. Firstly, Kaplan-Meier diagrams were plotted and the differences between groups were assessed with Gehan’s Wilcoxon test (GW). Next, its association with the age of incidence was verified by means of box plots and Mann-Whitney U test. Finally, the analysis of Cox’s proportional hazard was performed both as univariate analysis and as multivariate analysis adjusted for age. Spearman’s rank correlation test was applied for the assessment of the correlation between the age and the survival (only complete responses were included in this analysis).

## Results

### 1. Comparison of the Results Obtained with Real-Time PCR and FISH

FISH results were obtained for 37 cases (20 with normal EGFR gene copy number, 5 with polysomy, 5 with amplification and 7 with both polysomy and amplification), data in Table S2. The standard method gave true results in 27/37 cases (7/17 true positive and 20/20 true negative). The results obtained with the novel Real-Time PCR method were consistent with FISH results (both false positive and false negative detection rate for the novel method was equal to 0), and, therefore, were used for further analyses.

### 2. Analysis of the Clinical Aspects

**Figure 2:**
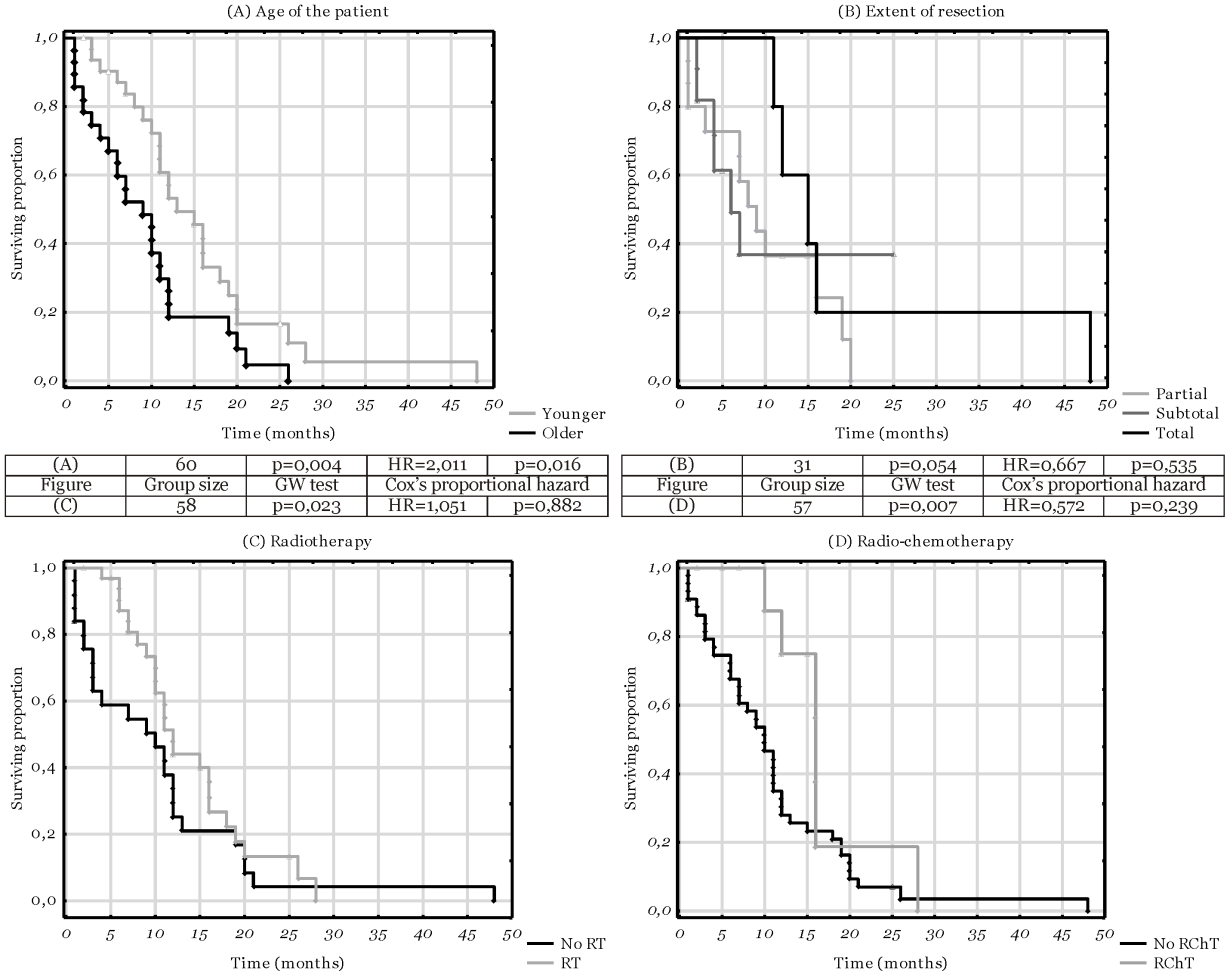
Kaplan-Meier diagrams depicting differences in survival times related to the clinical aspects. The attached table presents statistical data for each diagram. Cox's proportional hazard refers to univariate analysis for diagram A and to multivariate analysis for diagrams B, C, D. The calculated HR values pertain to the second subgroup listed (“total” subgroup for diagram B), while the HR values of the first subgroup (cumulatively of “partial” and “subtotal” subgroups for diagram B) equal to 1. ♦ - complete responses; Δ - censored responses. A. age of the patient, the threshold of 60 years included in the “younger” subgroup; B. extent of resection; C. radiotherapy; D. radio-chemotherapy.

Initially, the clinical data were evaluated in order to select the set of data for which to adjust the multivariate analyses. The significance of patient’s age was firstly assessed with the Spearman’s rank test. Spearman’s rho was -0.41 with p = 0.003. Next, an univariate analysis of Cox’s proportional hazard was performed both for age counted in years (Hazard Ratio, HR = 1.04; p = 0.0014) and in decades (HR = 1.41; p = 0.0056; Table 1, Fig. 2A). The analysis of the therapeutic process was performed both in the univariate and multivariate (adjusted for the age) manner. Albeit suggesting a possible effect with GW analysis (p = 0.20), the extent of resection appeared insignificant in the multivariate analysis (HR = 0.88, p = 0.67) (Fig. 2B). Neither did the comparison of gross total resection *vs.* the lower extents show any significant correlation with the outcome (p = 0.54). Next, the analysis of subsequent therapy showed that the patients treated with radiotherapy lived longer (p = 0.02), but also were significantly younger (p<0.01). The multivariate analysis showed no significance of the radiotherapy and attributed the differences in survival mostly to the age of the patient (p = 0.88) (Fig. 2C). Similarly, radio-chemotherapy was associated with both longer survival (p = 0.01) and younger age of the patients (p = 0.01), however, in the multivariate analysis a positive, yet insignificant, association was shown (HR = 0.57, p = 0.24) (data in Table 1, Fig. 2D). For the other analysed clinical data no significant correlation with the outcome was found (Table 1, complete data in Table S3).

**Table 1:** Selected results of the statistical analyses.

Criterion		Group size	GW Test	Cox’s Proportional Hazard	
**Clinical Data**					
**Age (years)**		60	NA	**HR = 1.042**	**p = 0.0014**
**Age (decades)**		60	NA	**HR = 1.409**	**p = 0.0056**
**Age (>60 y. o.)**		60	**p = 0.004**	**HR = 2.011**	**p = 0.016**
**Radiotherapy**		58	**p = 0.023**	HR = 1.051	p = 0.882
**Radio-chemotherapy**		57	**p = 0.007**	HR = 0.572	p = 0.239
**Extent of resection**		31	p = 0.199	HR = 0.879	p = 0.671
**Gross Total Resection**		31	p = 0.054	HR = 0.667	p = 0.535
**Molecular Data: direct analysis of the entire cohort**					
***TP53***		57	p = 0.279	HR = 0.949	p = 0.883
***EGFR***		59	p = 0.396	HR = 1.279	p = 0.456
**Poly 7**		57	p = 0.627	HR = 1.034	p = 0.920
***EGFRvIII***		58	**p = 0.037**	**HR = 0.337**	**p = 0.040**
***CDKN2A***		58	p = 0.180	HR = 0.774	p = 0.383
**Molecular Data: direct analysis of the age-dependant subgroups**					
***TP53***	**≤60 y. o.**	30	p = 0.144	HR = 0.636	p = 0.356
	**>60 y. o.**	27	p = 0.896	HR = 1.234	p = 0.683
***EGFR***	**≤60 y. o.**	32	**p = 0.006**	**HR = 3.745**	**p = 0.007**
	**>60 y. o.**	27	p = 0.300	HR = 0.601	p = 0.351
**Poly 7**	**≤60 y. o.**	30	p = 0.178	HR = 1.478	p = 0.388
	**>60 y. o.**	27	p = 0.544	HR = 0.646	p = 0.405
***EGFRvIII***	**≤60 y. o.**	31	p = 0.206	HR = 0.339	p = 0.150
	**>60 y. o.**	27	p = 0.107	HR = 0.198	p = 0.113
***CDKN2A***	**≤60 y. o.**	31	p = 0.441	HR = 0.922	p = 0.850
	**>60 y. o.**	27	p = 0.478	HR = 0.706	p = 0.479
**Molecular Data: the combinations of molecular characteristics**					
***EGFR*** ** amplified**	***TP53***	14	p = 0.937	HR = 1.127	p = 0.908
	**Poly 7**	15	**p = 0.049**	**HR = 14.879**	**p = 0.013**
	***EGFRvIII***	14	p = 0.078	HR = 0.094	p = 0.115
	***CDKN2A***	15	p = 0.010	HR = 0.119	p = 0.014
***EGFR*** ** non-amplified**	***TP53***	42	p = 0.387	HR = 1.121	p = 0.773
	**Poly 7**	42	p = 0.823	HR = 0.822	p = 0.614
	***EGFRvIII***	43	p = 0.199	HR = 0.420	p = 0.239
	***CDKN2A***	42	p = 0.849	HR = 1.026	p = 0.941
**Molecular Data: correlation with radiotherapy**					
**Radiotherapy**	***TP53***	30	p = 0.306	HR = 0.932	p = 0.879
	***EGFR***	33	**p = 0.022**	**HR = 2.713**	**p = 0.033**
	**Poly 7**	31	p = 0.238	HR = 1.309	p = 0.539
	***EGFRvIII***	32	p = 0.134	HR = 0.343	p = 0.155
	***CDKN2A***	32	p = 0.230	HR = 0.976	p = 0.955
**No Radiotherapy**	***TP53***	25	p = 0.670	HR = 0.827	p = 0.734
	***EGFR***	24	p = 0.903	HR = 1.535	p = 0.499
	**Poly 7**	24	p = 0.944	HR = 0.639	p = 0.436
	***EGFRvIII***	24	p = 0.548	HR = 0.561	p = 0.566
	***CDKN2A***	24	p = 0.627	HR = 0.749	p = 0.526

Cox’s Proprtional Hazard values pertain to the univariate analysis for age and to the multivariate analysis (adjusted for age) for other analyses. HR values refer to the presence of given feature.

For example:

In the group of patients younger than 60 years old, the risk of death over given time is 3.745 times higher in those with *EGFR* amplification than in those without the amplification.

Abbreviations:

*TP53*– *TP53* mutation;

*EGFR* – *EGFR* amplification;

Poly 7– chromosome 7 polysomy;

*EGFRvIII* – *EGFRvIII* expression;

*CDKN2A* – *CDKN2A* deletion;

y. o. – years old.

### 3. Analysis of the Molecular Aspects

#### 3.1. Direct analysis of the entire cohort

We observed the analysed alterations with the following frequencies: *TP53* mutation in 22% (17/79); *EGFR* amplification in 27% (22/82); chromosome 7 polysomy in 27% (21/79); *CDKN2A* deletion in 50% (40/80); *EGFRvIII* expression in 18% (14/80) and *IDH1* mutation in 3.9% (3/77).

**Figure 3:**
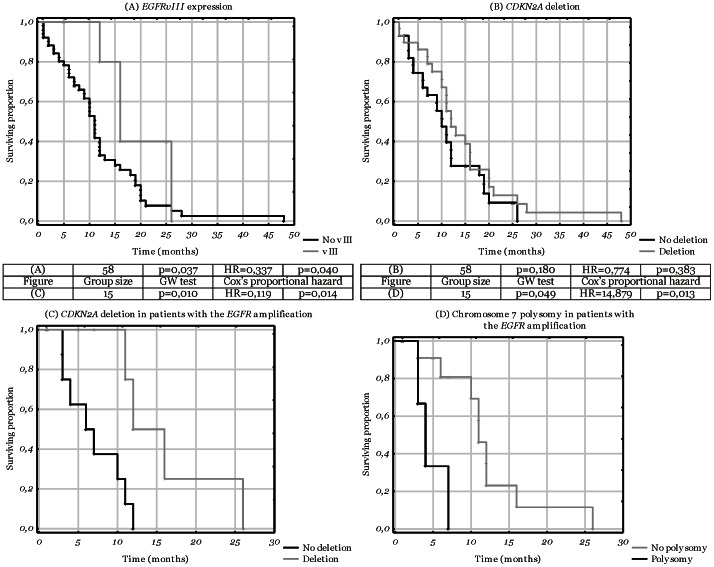
Kaplan-Meier diagrams depicting differences in survival times related to the molecular aspects. The attached table presents statistical data for each diagram. Cox’s proportional hazard refers to multivariate analysis. The calculated HR values pertain to the second subgroup listed, while the HR values of the first subgroup equal to 1. ♦ - complete responses; Δ - censored responses. A. *EGFRvIII* expression; B. *CDKN2A* deletion; C. The combination of *CDKN2A* deletion with *EGFR* amplification; D. the combination of chromosome 7 polysomy with *EGFR* amplification.

We did not observe any difference in the overall survival between the patients with and without *TP53* mutations in the direct analysis. We found neither *EGFR* amplification nor chromosome 7 polysomy to be prognostically significant. *EGFRvIII* expression appeared as a positive factor and although the GW test gave borderline result (p = 0.04), the multivariate analysis was confirmatory (HR = 0.34, p = 0.04) (Fig. 3A). Conversely, the significance of homozygous deletions of the *CDKN2A* gene, despite the suggestive Kaplan-Meier diagram (Fig. 3B), was negated both by GW test (p = 0.18) and by the multivariate analysis (p = 0.38). No statistical analyses were performed for the *IDH1* gene due to the low number of mutations.

#### 3.2. Analysis of age-dependant subgroups

**Figure 4:**
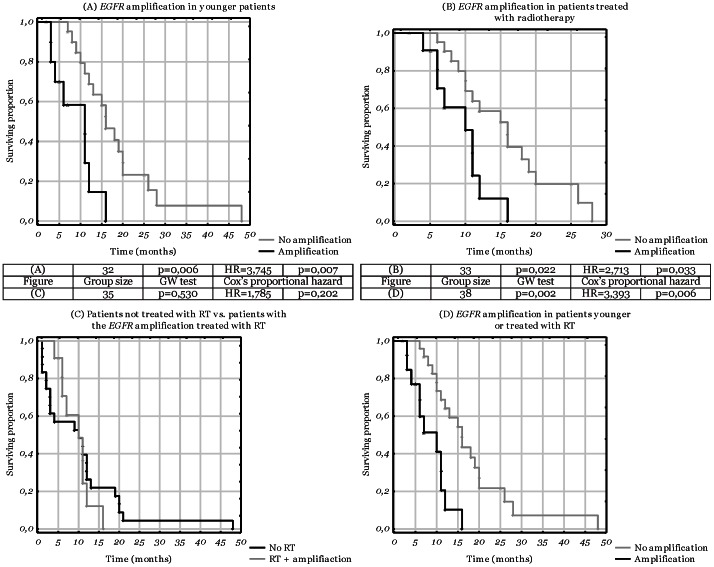
Kaplan-Meier diagrams depicting differences in survival times related to the *EGFR* amplification and clinical aspects. The attached table presents statistical data for each diagram. Cox’s proportional hazard refers to multivariate analysis. The calculated HR values pertain to the second subgroup listed, while the HR values of the first subgroup equal to 1. ♦ - complete responses; Δ - censored responses. A. *EGFR* amplification in patients aged 60 years and less; B. *EGFR* amplification in patients treated with radiotherapy; C. comparison of patients not treated with radiotherapy with those with the *EGFR* amplification treated with radiotherapy; D. *EGFR* amplification in a cumulative group of younger patients and those treated with radiotherapy.

In this part, the aforementioned analysis was performed in separate groups in relation to the age of the patients (with the threshold of 60 years, being the median age in the analysed group). Intriguingly, *EGFR* amplification appeared to have an opposite effect on survival in both groups. It seems to be associated with a shorter survival in younger patients and with a longer survival in older patients. Multivariate analysis confirmed the significance of *EGFR* amplification only in younger patients (HR = 3.75, p = 0.01) (Table 1, Fig. 4A).

#### 3.3. Analysis of combinations of molecular characteristics

We also analysed combinations of molecular alterations in relation with the survival. As mentioned before, homozygous deletions of *CDKN2A* were suspected to be related with a longer survival. Thus, it was intriguing to observe that such deletions were associated with an enhanced outcome in patients with *EGFR* amplification (HR = 0.12; p = 0.01)(Fig. 3C), while no difference was observed in patients without the amplification (p = 0.94). Next, the combination of both mechanism of the increased *EGFR* gene copy number appeared to be significant. In the group with the amplification the polysomy was associated with a poor outcome (HR = 14.88; p = 0.01; Fig. 3D), while no such association was observed in the group without the amplification (p = 0.61; Table 1).

#### 3.4. Correlation with the therapy applied

We evaluated the association between the molecular characteristics and the effectiveness of radiotherapy (reflected by the overall survival) and the only divergence was observed for *EGFR* amplification. In patients who did not receive radiotherapy the *EGFR* amplification did not influence the survival (p = 0.50), while in patients who were treated with radiotherapy it was related to an impaired survival (HR = 2.71; p = 0.03) (Fig. 4B). Intriguingly, we did not observe any differences between the patients with *EGFR* amplification treated with radiotherapy and the patients who had not received radiotherapy (irrespective of the *EGFR* status) (p = 0.20) (Fig. 4C). To investigate the correlation between the amplification and the survival in younger patients and those treated with radiotherapy two additional analyses were performed – one in a cumulative group of younger patients and those treated with radiotherapy (HR = 3.39; p = 0.01; Fig. 4D) and the other only in patients fulfilling both criteria simultaneously (HR = 2.78; p = 0.06). A similar analysis for the chemotherapy was not conducted due to the small group of patients who underwent such a treatment.

## Discussion

The presented technique for the *EGFR* gene overdosage analysis allows for a clear discrimination between *EGFR* amplification and chromosome 7 polysomy. The outspoken need for a simple method serving such purposes was expressed in several publications [19], [20], [21]. The verification of the results proved the method to be highly reliable (both false negative and false positive detection rate were equal to 0 in this group, however, it cannot be excluded that false results may occur in a more numerous group or as a result of low quality DNA, lower tumour cell content or technical errors, etc.). Until now, such an approach for the detection and discrimination between the *EGFR* amplification and chromosome 7 polysomy with Real-Time PCR at the DNA level was not described. The commonly applied methods for *EGFR* gene copy number assessment at the DNA level are based on the ratio of *EGFR* to the reference, with the threshold for the amplification’s recognition ranged from more than 1 to more than 5, while chromosome 7 polysomy was rarely taken into account [14], [15], [22], [23], [24]. The analysed interpretation of the standard Real-Time PCR (the ratio of *EGFR* to the reference gene between 1.5 and 5 is considered as polysomy, while a ratio higher than 5 is indicative of amplification) allows for the detection and the discrimination between both mechanisms of the increased *EGFR* gene copy number with a limited precision. Such an approach is effective in some cases, but it can lead to confusing results in 3 situations: the amplification present in a low proportion of cells or a low-magnitude amplification (mistaken for the polysomy), a high-magnitude polysomy (mistaken for the amplification) and the coincidence of amplification and polysomy (polysomy not detected). The proposed technique was efficient in the recognition of each of these situations. Obviously, it is not our suggestion that this method may replace FISH at its position as the golden standard, particularly due to the fact that it allows for the detection of the alterations at a single cell level. Nevertheless, the presented technique allows for the reliable as well as time- and cost-effective analysis of the general population of cells at DNA level, which makes it an attractive possibility for the screening of the *EGFR* overdosage.

The frequencies of the observed alterations were in general similar to the literature data. In the analysed group, the frequency of *IDH1* mutation was in accordance with the analysis by Nobusawa *et al.* (3.9% *vs.* 3.7%) [25]. The analysed group was characterised by an overrepresentation of *CDKN2A* deletions (50% *vs.* 31%) in comparison to the analysis by Ohgaki *et al.* [1]. Other markers were underrepresented in the analysed group. The frequency of *TP53* mutation was 23% (*vs.* 28%), of *EGFR* amplification 27% (*vs.* 36%) [1]; of *EGFRvIII* expression 18% (*vs.* 27% or 31% [26], [27]) and of chromosome 7 polysomy was 27% (*vs.* 39% [28]).

In the clinical data, the age of a patient was a significant prognostic factor in accordance with the published data [7], [29], [30]. Two other factors (KPS and RTOG classification), described in many reports as significantly correlated with the outcome [7], [30], could not be assessed in our analysis. The issue of the significance of the extent of resection remains discordant in the literature, either marking it as a factor of great importance [5], [31] or diminishing its role [29], [30]; our data concur with the latter. Albeit expected and suggested in the literature [2], [3], [32], the therapy given after surgery did not prove to significantly improve the survival of patients when the analyses were adjusted for age.

To date, *EGFRvIII* expression has been associated either with poorer prognosis of glioblastoma (in combination with the amplification) [29] or, most commonly, no association has been observed [26], [27], [33]. Nonetheless, Liu *et al.* suggested a correlation of the *EGFRvIII* expression and a longer survival in anaplastic astrocytoma patients [27]. Thus far, only the report by Montano *et al.* [7] has shown an association of the *EGFRvIII* expression with a more favourable prognosis of glioblastoma, which the results presented here support.

The possible prognostic value of *EGFR* amplification and overexpression has been intensively analysed, in most cases without any significant correlation with the clinical outcome observed [26], [27], [31], [33], [34], [35]. However, Simmons *et al*. observed that *EGFR* overexpression was differently correlated with survival in separate age groups (with the threshold of 55 years), *i.e.* the overexpression indicated worse prognosis in younger patients and better prognosis in older patients [30], which concurs with the results of our analysis. On the other hand, the analysis by Shinojima *et al.* suggested that *EGFR* amplification was associated with poorer prognosis in the entire cohort with an emphasis on younger patients [29]. The analysis of patients younger than 50 years old by Korshunov *et al.* indicated a negative influence of amplification on survival [36]. Our data suggest that *EGFR* amplification may be related to a worse prognosis in younger patients and in patients treated with radiotherapy, while improving the prognosis in older patients. Bearing in mind the strong correlation between the age and treatment with radiotherapy (p<0.01), it should be verified which factor is the leading one. The analyses of the cumulative group (younger or treated with radiotherapy) and of only younger patients treated with radiotherapy may imply that *EGFR* amplification may be a negative prognostic factor for patients whose survival is not limited by their overall clinical condition. If verified, these observations may emphasise the need for a therapy specifically targeting the EGFR pathway in this group of patients. Nonetheless, the analysed group in this study is not large enough for such far-reaching conclusions. An association between *EGFR* amplification and the effectiveness of the radiotherapy requires a thorough verification, the more so that it may potentially affect the selection of treatment in future. To date, however, the analysis of Ang *et al*. suggested that the amplification was related to a better outcome in patients treated with radio-chemotherapy [37].

The prognostic significance of chromosome 7 polysomy has not been extensively analysed in glioblastoma. Our data suggest that the combination of *EGFR* amplification and polysomy may be correlated with poor prognosis, which requires further verification. Moreover, our data also indicate that both mechanisms of the increased *EGFR* gene copy number (polysomy and amplification) need to be distinguished and analysed separately.

A prognostic significance of homozygous deletions of *CDKN2A* has not been validated thus far. Amongst the published reports, every possible correlation between the deletion and the outcome (positive [38], negative [39] or no correlation [31], [40]) has been observed. Homozygous deletion of *CDKN2A* was associated with an improved outcome in patients with the *EGFR* amplification, but not in patients without this alteration. Nonetheless, our data do not allow to unquestionably verify whether this correlation was random or specific.

The reports analysing *TP53* mutations in glioblastoma unanimously recognise its lack of prognostic significance in the general population [5], [30], [31], [35], [41]. On the other hand, Ruano *et al.* proposed that simultaneous *TP53* mutation and *EGFR* amplification may be related to a poorer prognosis [41]. Conversely, in the analysis by Simmons *et al.* the two cases with the concurrent *TP53* mutation and *EGFR* overexpression were characterised by a relatively long survival [30]. The only patient with the simultaneous alterations in this report had survived 11 months from the diagnosis (which was approximately the median survival time). In the report by Simmons *et al.* the *TP53* mutation was somewhat related to a shorter survival (not significant) in the group of patients without the *EGFR* amplification [30].

A positive prognostic significance of the *IDH1* mutation has been suggested by several authors [25], [42] due to its relation to the secondary glioblastoma, which are generally characterised by a more favourable outcome [1], however, we could not perform any reliable analysis due to the low number of such mutations.

### Conclusion

To conclude, the presented method was efficient and reliable in detection and distinction between *EGFR* amplification and chromosome 7 polysomy. *EGFR* amplification was identified as a factor significantly limiting the effectiveness of radiotherapy and the survival of younger patients. Although the presented data are not sufficient to question the indications for radiotherapy for glioblastoma patients with *EGFR* amplification and require validation in a larger group of patients, they strongly advocate for the consideration of the patient’s molecular status in the putative selection of the therapy, especially in the light of the numerous novel therapeutic possibilities being introduced.

## Supporting Information

Figure S1 **Exemplary results of FISH and TP53 sequencing.** A. Exemplary FISH result presenting both *EGFR* amplification and chromosome 7 polysomy; magnification 1000x, *EGFR* signals are red, CEP7 signals are green, scaling bar marks 10 µm. B. Exemplary FISH in FFPE sample result presenting *EGFR* amplification; magnification 1000x, *EGFR* signals are red, CEP7 signals are green, scaling bar marks 10 µm. C. Exemplary FISH in FFPE sample result presenting *EGFR* amplification; magnification 600x, *EGFR* signals are red, CEP7 signals are green, scaling bar marks 10 µm. D. Exemplary *TP53* sequencing result, an arrow marks the mutated nucleotide in codon 237.
(TIF)

Table S1 **Primer sequences.** ^a^ – For the sequencing of IDH1 two sense primers were used.
(DOC)

Table S2 **Comparison of the results obtained with FISH with those obtained with the standard and novel Real-Time PCR based methods.** For the standard method the ratios of EGFR to RNaseP and their interpretations (<1.5– normal; 1.5–5– polysomy; >5– amplification) are presented. For the novel method the ratios of EGFR to RNaseP, EGFR to GPER and GPER to RNaseP and their combined interpretations (EGFR/GPER >1.5– amplification; GPER/RNaseP>1.5 - polysomy) are presented.
(DOC)

Table S3 **Complete results of the statistical analyses.** Cox’s Proprtional Hazard values pertain to the univariate analysis for age and to the multivariate analysis (adjusted for age) for other analyses. HR values refer to the presence of given feature. For example: In the group of patients younger than 60 years old, the risk of death over given time is 3.745 times higher in those with EGFR amplification than in those without the amplification. Abbreviations: TP53– TP53 mutation; EGFR – EGFR amplification; Poly 7– chromosome 7 polysomy; EGFRvIII – EGFRvIII expression; CDKN2A – CDKN2A deletion; y. o. – years old ^a^ – Invasion of given location by the tumour.
(DOC)
