# Supplementary figures and images for: Screening for EGFR Amplifications with a Novel Method and Their Significance for the Outcome of Glioblastoma Patients
Source: PLoS One. 2013 Jun 6;8(6):e65444. doi: 10.1371/journal.pone.0065444 (PMC3675194; doi:10.1371/journal.pone.0065444)

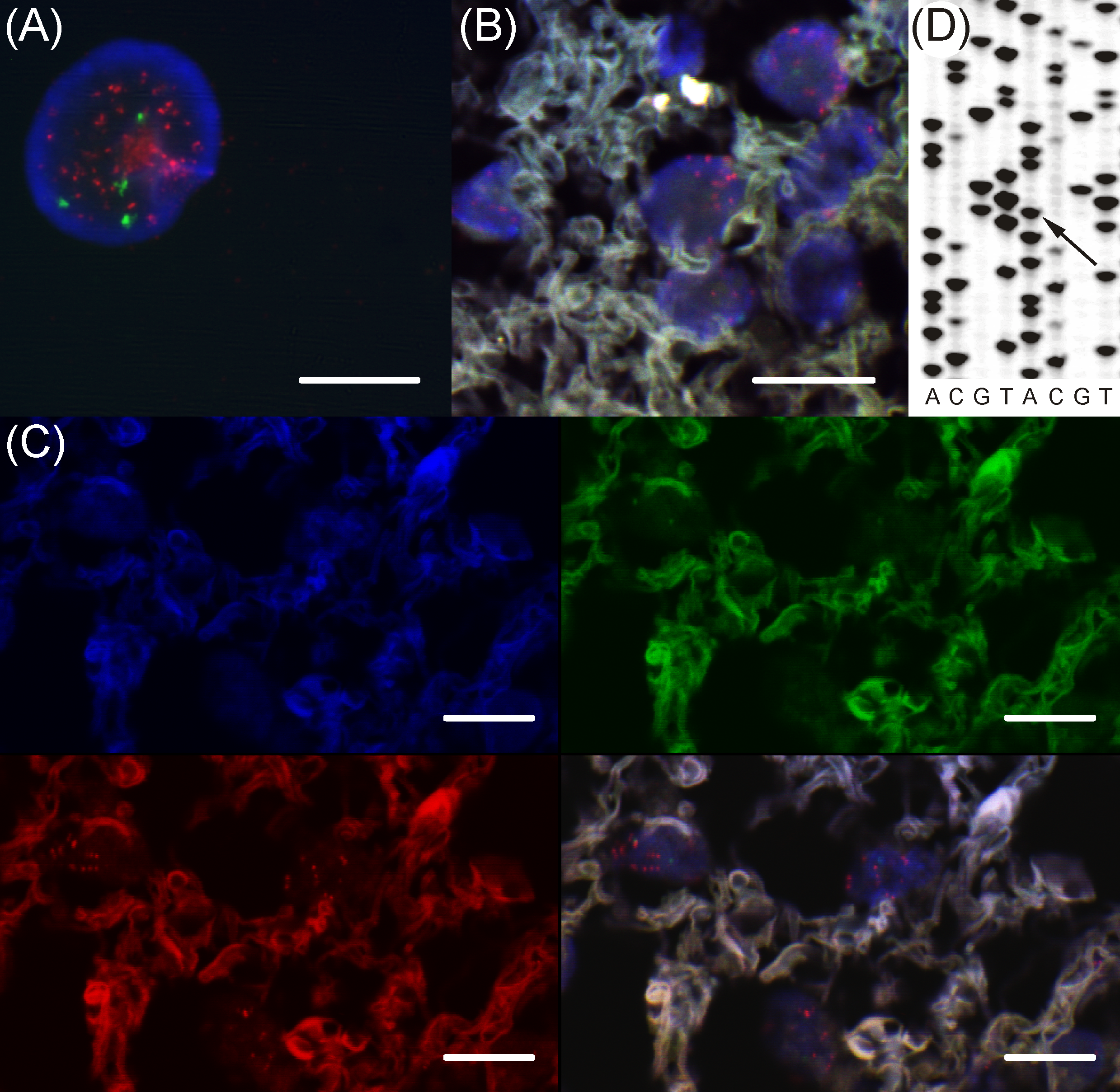

Supplement: Figure S1 — Exemplary results of FISH and TP53 sequencing. A. Exemplary FISH result presenting both EGFR amplification and chromosome 7 polysomy; magnification 1000x, EGFR signals are red, CEP7 signals are green, scaling bar marks 10 µm. B. Exemplary FISH in FFPE sample result presenting EGFR amplification; magnification 1000x, EGFR signals are red, CEP7 signals are green, scaling bar marks 10 µm. C. Exemplary FISH in FFPE sample result presenting EGFR amplification; magnification 600x, EGFR signals are red, CEP7 signals are green, scaling bar marks 10 µm. D. Exemplary TP53 sequencing result, an arrow marks the mutated nucleotide in codon 237. (TIF) [file pone.0065444.s001.tif]
