# Supplementary material for: Screening for EGFR Amplifications with a Novel Method and Their Significance for the Outcome of Glioblastoma Patients
Source: PLoS One. 2013 Jun 6;8(6):e65444. doi: 10.1371/journal.pone.0065444 (PMC3675194; doi:10.1371/journal.pone.0065444)
Supplement: Table S1 — Primer sequences. a – For the sequencing of IDH1 two sense primers were used. (DOC) [file pone.0065444.s002.doc]

Tab. S1. Primer sequences.

| **Gene** | **Sense primer** | **Antisense primer** |
| --- | --- | --- |
| ***EGFR*** | 5'-CACACCCCTGACTCTCCACT-3' | 5'-GAGACAATCCTGTGAGCTTGG-3' |
| ***GPER*** | 5'-CATCTGGACGGCAGGTAAGT-3' | 5'-CCCTCAGCCGGTAGTTTTC-3' |
| ***RNase*** | 5'-GGGAGATGCGGAAGAATGT-3' | 5'-CCTCCAGTCAGCCACAGAA-3' |
| ***TP53* (PCR)** | 5'-GTGCAGCTGTGGGTTGATT-3' | 5'-GCAGTGCTCGCTTAGTGCTC-3' |
| ***TP53* (Sequencing)** | 5'-GCCATCTACAAGCAGTCACA-3' | 5'-CCCTTTCTTGCGGAGATTCT-3' |
| ***IDH1* (PCR)** | 5'-GGCACCCATCTTCTGTGTTT-3' | 5'-ATATGCATTTCTCAATTTCAT-3' |
| ***IDH1* (Sequencing)^a^** | 5'-GCAAAAATATCCCCCGGCTT-3' | – |
|  | 5'-CGGTCTTCAGAGAAGCCATT-3' | – |
| ***CDKN2A* exon 1** | 5'-CAACGCACCGAATAGTTACG-3' | 5'-CTGCAAACTTCGTCCTCCAG-3' |
| ***CDKN2A* exon 2** | 5'-ACCAGAGGCAGTAACCATGC-3' | 5'-TGGAAGCTCTCAGGGTACAAA-3' |
| ***EGFRvIII*** | 5'-GGCTCTGGAGGAAAAGAAAGGTAAT-3' | 5'-TCCTCCATCTCATAGCTGTCG-3' |
| ***GUSB*** | 5'-CTCATTTGGAATTTTGCCGATT-3' | 5'-CCGAGTGAAGATCCCCTTTTTA-3' |

^a^ – For the sequencing of *IDH1* two sense primers were used.
