# Supplementary material for: Screening for EGFR Amplifications with a Novel Method and Their Significance for the Outcome of Glioblastoma Patients
Source: PLoS One. 2013 Jun 6;8(6):e65444. doi: 10.1371/journal.pone.0065444 (PMC3675194; doi:10.1371/journal.pone.0065444)
Supplement: Table S2 — Comparison of the results obtained with FISH with those obtained with the standard and novel Real-Time PCR based methods. For the standard method the ratios of EGFR to RNaseP and their interpretations (<1.5– normal; 1.5–5– polysomy; >5– amplification) are presented. For the novel method the ratios of EGFR to RNaseP, EGFR to GPER and GPER to RNaseP and their combined interpretations (EGFR/GPER >1.5– amplification; GPER/RNaseP>1.5 - polysomy) are presented. (DOC) [file pone.0065444.s003.doc]

Tab. S2. Comparison of the results obtained with FISH with those obtained with the standard and novel Real-Time PCR based methods.

| **Case no.** | **Standard Real-Time PCR** | | **Novel Real-Time PCR** | | | | **FISH** |
| --- | --- | --- | --- | --- | --- | --- | --- |
|  | ***EGFR*/*RNaseP*** | **Interpretation** | ***EGFR*/*RNaseP*** | ***EGFR*/*GPER*** | ***GPER*/*RNaseP*** | **Interpretation** |  |
| 1 | 12,33 | Amplification | 12,33 | 4,47 | 2,76 | Amplification and polysomy | Amplification and polysomy |
| 2 | 2,52 | Polysomy | 2,52 | 1,30 | 1,94 | Polysomy | Polysomy |
| 3 | 3,56 | Polysomy | 3,56 | 1,72 | 2,07 | Amplification and polysomy | Amplification and polysomy |
| 4 | 8,40 | Amplification | 8,40 | 0,79 | 10,65 | Polysomy | Polysomy |
| 5 | 2,45 | Polysomy | 2,45 | 1,08 | 2,28 | Polysomy | Polysomy |
| 6 | 2,60 | Polysomy | 2,60 | 1,07 | 2,43 | Polysomy | Polysomy |
| 7 | 11,86 | Amplification | 11,86 | 10,93 | 1,08 | Amplification | Amplification |
| 8 | 6,67 | Amplification | 6,67 | 9,29 | 0,72 | Amplification | Amplification |
| 9 | 20,15 | Amplification | 20,15 | 7,11 | 2,83 | Amplification and polysomy | Amplification and polysomy |
| 10 | 3,45 | Polysomy | 3,45 | 1,61 | 2,14 | Amplification and polysomy | Amplification and polysomy |
| 11 | 4,45 | Polysomy | 4,45 | 6,00 | 0,74 | Amplification | Amplification |
| 12 | 34,35 | Amplification | 34,35 | 13,63 | 2,52 | Amplification and polysomy | Amplification and polysomy |
| 13 | 103,49 | Amplification | 103,49 | 28,84 | 3,59 | Amplification and polysomy | Amplification and polysomy |
| 14 | 10,48 | Amplification | 10,48 | 9,14 | 1,15 | Amplification | Amplification |
| 15 | 2,10 | Polysomy | 2,10 | 1,14 | 1,85 | Polysomy | Polysomy |
| 16 | 4,78 | Polysomy | 4,78 | 4,65 | 1,03 | Amplification | Amplification |
| 17 | 9,90 | Amplification | 9,90 | 3,97 | 2,49 | Amplification and polysomy | Amplification and polysomy |
| 18 | 1,23 | Normal | 1,23 | 0,87 | 1,41 | Normal | Normal |
| 19 | 1,09 | Normal | 1,09 | 0,83 | 1,32 | Normal | Normal |
| 20 | 0,77 | Normal | 0,77 | 0,82 | 0,94 | Normal | Normal |
| 21 | 0,99 | Normal | 0,99 | 0,82 | 1,22 | Normal | Normal |
| 22 | 1,13 | Normal | 1,13 | 1,00 | 1,14 | Normal | Normal |
| 23 | 1,47 | Normal | 1,47 | 1,19 | 1,23 | Normal | Normal |
| 24 | 1,25 | Normal | 1,25 | 1,12 | 1,12 | Normal | Normal |
| 25 | 1,26 | Normal | 1,26 | 1,04 | 1,21 | Normal | Normal |
| 26 | 0,76 | Normal | 0,76 | 0,83 | 0,91 | Normal | Normal |
| 27 | 0,90 | Normal | 0,90 | 0,90 | 1,00 | Normal | Normal |
| 28 | 0,73 | Normal | 0,73 | 0,97 | 0,74 | Normal | Normal |
| 29 | 1,15 | Normal | 1,15 | 0,90 | 1,28 | Normal | Normal |
| 30 | 0,82 | Normal | 0,82 | 0,70 | 1,17 | Normal | Normal |
| 31 | 0,66 | Normal | 0,66 | 0,82 | 0,80 | Normal | Normal |
| 32 | 1,11 | Normal | 1,11 | 0,81 | 1,37 | Normal | Normal |
| 33 | 0,98 | Normal | 0,98 | 0,80 | 1,24 | Normal | Normal |
| 34 | 1,23 | Normal | 1,23 | 1,24 | 0,99 | Normal | Normal |
| 35 | 0,91 | Normal | 0,91 | 0,72 | 1,25 | Normal | Normal |
| 36 | 0,57 | Normal | 0,57 | 0,71 | 0,81 | Normal | Normal |
| 37 | 0,84 | Normal | 0,84 | 0,94 | 0,89 | Normal | Normal |

For the standard method the ratios of *EGFR* to *RNaseP* and their interpretations (<1.5 – normal; 1.5-5 – polysomy; >5 – amplification) are presented. For the novel method the ratios of *EGFR* to *RNaseP*, *EGFR* to *GPER* and *GPER* to *RNaseP* and their combined interpretations (*EGFR*/*GPER* > 1.5 – amplification; *GPER*/*RNaseP* > 1.5 - polysomy) are presented.
