# Supplementary material for: Screening for EGFR Amplifications with a Novel Method and Their Significance for the Outcome of Glioblastoma Patients
Source: PLoS One. 2013 Jun 6;8(6):e65444. doi: 10.1371/journal.pone.0065444 (PMC3675194; doi:10.1371/journal.pone.0065444)
Supplement: Table S3 — Complete results of the statistical analyses. Cox’s Proprtional Hazard values pertain to the univariate analysis for age and to the multivariate analysis (adjusted for age) for other analyses. HR values refer to the presence of given feature. For example: In the group of patients younger than 60 years old, the risk of death over given time is 3.745 times higher in those with EGFR amplification than in those without the amplification. Abbreviations: TP53– TP53 mutation; EGFR – EGFR amplification; Poly 7– chromosome 7 polysomy; EGFRvIII – EGFRvIII expression; CDKN2A – CDKN2A deletion; y. o. – years old a – Invasion of given location by the tumour. (DOC) [file pone.0065444.s004.doc]

Tab. S3. Complete results of the statistical analyses.

| **Criterion** | | **Group size** | **GW Test** | **Cox’s Proportional Hazard** | |
| --- | --- | --- | --- | --- | --- |
| **Clinical Data** | | | | | |
| **Age (years)** | | 60 | NA | **HR = 1,042** | **p = 0,0014** |
| **Age (decades)** | | 60 | NA | **HR = 1,409** | **p = 0,0056** |
| **Age (>60 y. o.)** | | 60 | **p = 0,004** | **HR = 2,011** | **p = 0,016** |
| **Sex** | | 60 | p = 0,181 | HR = 1,019 | p = 0,950 |
| **Radiotherapy** | | 58 | **p = 0,023** | HR = 1,051 | p = 0,882 |
| **Radio-chemotherapy** | | 57 | **p = 0,007** | HR = 0,572 | p = 0,239 |
| **Extent of resection** | | 31 | p = 0,199 | HR = 0,879 | p = 0,671 |
| **Gross Total Resection** | | 31 | p = 0,054 | HR = 0,667 | p = 0,535 |
| **Frontal location^a^** | | 56 | p = 0,715 | HR = 1,222 | p = 0,527 |
| **Temporal location^a^** | | 56 | p = 0,546 | HR = 0,814 | p = 0,514 |
| **Occipital location^a^** | | 56 | p = 0,428 | HR = 1,319 | p = 0,493 |
| **Parietal location^a^** | | 56 | p = 0,482 | HR = 0,829 | p = 0,548 |
| **2 or more lobes affected** | | 56 | p = 0,864 | HR = 0,963 | p = 0,904 |
| **Molecular Data: direct analysis of the entire cohort** | | | | | |
| ***TP53*** | | 57 | p = 0,279 | HR = 0,949 | p = 0,883 |
| ***EGFR*** | | 59 | p = 0,396 | HR = 1,279 | p = 0,456 |
| **Poly 7** | | 57 | p = 0,627 | HR = 1,034 | p = 0,920 |
| ***EGFRvIII*** | | 58 | **p = 0,037** | **HR = 0,337** | **p = 0,040** |
| ***CDKN2A*** | | 58 | p = 0,180 | HR = 0,774 | p = 0,383 |
| **Molecular Data: direct analysis of the age-dependant subgroups** | | | | | |
| ***TP53*** | **≤60 y. o.** | 30 | p = 0,144 | HR = 0,636 | p = 0,356 |
|  | **>60 y. o.** | 27 | p = 0,896 | HR = 1,234 | p = 0,683 |
| ***EGFR*** | **≤60 y. o.** | 32 | **p = 0,006** | **HR = 3,745** | **p = 0,007** |
|  | **>60 y. o.** | 27 | p = 0,300 | HR = 0,601 | p = 0,351 |
| **Poly 7** | **≤60 y. o.** | 30 | p = 0,178 | HR = 1,478 | p = 0,388 |
|  | **>60 y. o.** | 27 | p = 0,544 | HR = 0,646 | p = 0,405 |
| ***EGFRvIII*** | **≤60 y. o.** | 31 | p = 0,206 | HR = 0,339 | p = 0,150 |
|  | **>60 y. o.** | 27 | p = 0,107 | HR = 0,198 | p = 0,113 |
| ***CDKN2A*** | **≤60 y. o.** | 31 | p = 0,441 | HR = 0,922 | p = 0,850 |
|  | **>60 y. o.** | 27 | p = 0,478 | HR = 0,706 | p = 0,479 |
| **Molecular Data: the combinations of molecular characteristics** | | | | | |
| ***TP53* mutated** | ***EGFR*** | 14 | p = 0,705 | HR = 2,633 | p = 0,391 |
|  | **Poly 7** | 14 | p = 0,614 | HR = 0,600 | p = 0,493 |
|  | ***EGFRvIII*** | 14 | p = 0,705 | HR = 1,768 | p = 0,620 |
|  | ***CDKN2A*** | 13 | p = 0,453 | HR = 1,484 | p = 0,606 |
| ***TP53* wild type** | ***EGFR*** | 42 | p = 0,926 | HR = 1,098 | p = 0,808 |
|  | **Poly 7** | 40 | p = 0,992 | HR = 0,966 | p = 0,934 |
|  | ***EGFRvIII*** | 42 | **p = 0,043** | **HR = 0,248** | **p = 0,027** |
|  | ***CDKN2A*** | 42 | p = 0,547 | HR = 0,783 | p = 0,477 |
| ***EGFR* amplified** | ***TP53*** | 14 | p = 0,937 | HR = 1,127 | p = 0,908 |
|  | **Poly 7** | 15 | **p = 0,049** | **HR = 14,879** | **p = 0,013** |
|  | ***EGFRvIII*** | 14 | p = 0,078 | HR = 0,094 | p = 0,115 |
|  | ***CDKN2A*** | 15 | **p = 0,010** | **HR = 0,119** | **p = 0,014** |
| ***EGFR* non-amplified** | ***TP53*** | 42 | p = 0,387 | HR = 1,121 | p = 0,773 |
|  | **Poly 7** | 42 | p = 0,823 | HR = 0,822 | p = 0,614 |
|  | ***EGFRvIII*** | 43 | p = 0,199 | HR = 0,420 | p = 0,239 |
|  | ***CDKN2A*** | 42 | p = 0,849 | HR = 1,026 | p = 0,941 |
| **Polysomy 7** | ***TP53*** | 12 | p = 1,000 | HR = 1,053 | p = 0,936 |
|  | ***EGFR*** | 14 | p = 0,087 | HR = 9,501 | p = 0,056 |
|  | ***EGFRvIII*** | 12 | p = 0,904 | HR = 0,755 | p = 0,779 |
|  | ***CDKN2A*** | 14 | p = 0,938 | HR = 1,475 | p = 0,591 |
| **No polysomy** | ***TP53*** | 42 | p = 0,381 | HR = 1,177 | p = 0,730 |
|  | ***EGFR*** | 43 | p = 0,901 | HR = 0,975 | p = 0,950 |
|  | ***EGFRvIII*** | 43 | **p = 0,031** | HR = 0,289 | p = 0,050 |
|  | ***CDKN2A*** | 42 | p = 0,117 | HR = 0,613 | p = 0,175 |
| ***EGFRvIII*** | ***TP53*** | 7 | p = 0,826 | HR = 2,320 | p = 0,649 |
|  | ***EGFR*** | 7 | p = 0,640 | HR = 0,566 | p = 0,650 |
|  | **poly 7** | 7 | p = 0,232 | HR = 1,254 | p = 0,546 |
|  | ***CDKN2A*** | 7 | p = 0,876 | HR = 0,792 | p = 0,864 |
| **No  *EGFRvIII*** | ***TP53*** | 49 | p = 0,215 | HR = 0,793 | p = 0,531 |
|  | ***EGFR*** | 50 | p = 0,335 | HR = 1,986 | p = 0,082 |
|  | **poly 7** | 48 | p = 0,805 | HR = 0,829 | p = 0,608 |
|  | ***CDKN2A*** | 49 | p = 0,332 | HR = 0,758 | p = 0,377 |
| ***CDKN2A* deletion** | ***TP53*** | 28 | p = 0,231 | HR = 1,354 | p = 0,586 |
|  | ***EGFR*** | 28 | p = 0,359 | HR = 0,630 | p = 0,417 |
|  | **poly 7** | 27 | p = 0,532 | HR = 1,495 | p = 0,469 |
|  | ***EGFRvIII*** | 29 | p = 0,128 | HR = 0,430 | p = 0,201 |
| ***CDKN2A* retention** | ***TP53*** | 27 | p = 0,696 | HR = 0,940 | p = 0,911 |
|  | ***EGFR*** | 29 | p = 0,124 | HR = 2,223 | p = 0,095 |
|  | **poly 7** | 29 | p = 0,757 | HR = 0,648 | p = 0,364 |
|  | ***EGFRvIII*** | 27 | p = 0,203 | HR = 0,225 | p = 0,130 |
| **Molecular Data: correlation with radiotherapy** | | | | | |
| **Radiotherapy** | ***TP53*** | 30 | p = 0,306 | HR = 0,932 | p = 0,879 |
|  | ***EGFR*** | 33 | **p = 0,022** | **HR = 2,713** | **p = 0,033** |
|  | **Poly 7** | 31 | p = 0,238 | HR = 1,309 | p = 0,539 |
|  | ***EGFRvIII*** | 32 | p = 0,134 | HR = 0,343 | p = 0,155 |
|  | ***CDKN2A*** | 32 | p = 0,230 | HR = 0,976 | p = 0,955 |
| **No Radiotherapy** | ***TP53*** | 25 | p = 0,670 | HR = 0,827 | p = 0,734 |
|  | ***EGFR*** | 24 | p = 0,903 | HR = 1,535 | p = 0,499 |
|  | **Poly 7** | 24 | p = 0,944 | HR = 0,639 | p = 0,436 |
|  | ***EGFRvIII*** | 24 | p = 0,548 | HR = 0,561 | p = 0,566 |
|  | ***CDKN2A*** | 24 | p = 0,627 | HR = 0,749 | p = 0,526 |

Cox’s Proprtional Hazard values pertain to the univariate analysis for age and to the multivariate analysis (adjusted for age) for other analyses.
HR values refer to the presence of given feature.

For example: In the group of patients younger than 60 years old, the risk of death over given time is 3.745 times higher in those with *EGFR* amplification than in those without the amplification.

Abbreviations:

*TP53* – *TP53* mutation;

*EGFR* – *EGFR* amplification;

Poly 7 – chromosome 7 polysomy;

*EGFRvIII* – *EGFRvIII* expression;

*CDKN2A* – *CDKN2A* deletion;

y. o. – years old

^a^ – Invasion of given location by the tumour.
